# Qualitative drivers of postoperative prophylactic antibiotics use and resistance in Ethiopia

**DOI:** 10.1186/s12913-024-11650-4

**Published:** 2024-10-22

**Authors:** Samantha Steeman, Maia R. Nofal, Ibrahim Heyredin, Hailemichael Asmamaw, Assefa Tesfaye, Alex Zhuang, Natnael Gebeyehu, Sylvia Bereknyei Merrell, Thomas G. Weiser, Tihitena Negussie Mammo

**Affiliations:** 1 Stanford University School of Medicine, Stanford, CA USA; 2 Boston Medical Center, Department of Surgery, Boston University Chobanian & Avedisian School of Medicine, Boston, MA USA; 3 Department of Surgery, Stanford University, Stanford, CA USA; 4 Lifebox, Addis Ababa, Ethiopia; 5 Fogarty International Center, Global Health Equity Scholars Program (D43TW010540), Washington, D.C USA; 6 St. Peter’s Specialized Hospital, Addis Ababa, Ethiopia; 7 Black Lion Specialized Hospital, Addis Ababa, Ethiopia; 8 Boston University Chobanian & Avedisian School of Medicine, Boston, MA USA; 9 Fogarty International Center, Harvard-BU-Northwestern-UNM Consortium (D43TW010543), Washington, D.C USA; 10 Addis Ababa University College of Health Science, Addis Ababa, Ethiopia; 11 Department of Pediatrics, Office of Child Health Equity, Palo Alto, CA USA; 12 Department of Surgery, Stanford-Surgery Policy Improvement Research & Education Center (S-SPIRE), Palo Alto, CA USA; 13 Boston University Chobanian & Avedisian School of Medicine Surgery Education Office Boston Medical Center, 85 E. Concord Street, Third Floor, Boston, MA 02118 USA

**Keywords:** Ethiopia, Postoperative prophylaxis, Antibiotics resistance, Surgical site infection

## Abstract

**Background:**

The World Health Organization (WHO) cautions against unnecessary prolongation of postoperative antibiotics to prevent surgical site infections (SSI), however this practice is still common in many countries. This study aims to describe drivers of prolonged postoperative antibiotic prescribing and clinicians’ perspectives on antibiotics resistance and stewardship in Ethiopia.

**Methods:**

We conducted semi-structured interviews of 16 surgeons and nine surgical ward nurses at three academic referral hospitals in Addis Ababa. Audio recordings were transcribed verbatim and coded. Codes were inductively and iteratively derived between two researchers, tested for inter-rater reliability (IRR), and the codebook was consistently applied to all transcripts. Thematic analysis was performed to understand drivers of prolonged prophylactic antibiotic use in surgical patients.

**Results:**

Interviews revealed factors contributing to postoperative prophylactic antibiotics overprescribing, including inadequate infection prevention and control (IPC) practices, wide variability in local prescribing practices, and distrust in the applicability of WHO guidelines. Antimicrobial resistance was also identified as a major concern by staff. Barriers to improving stewardship included a lack of multidisciplinary teamwork to inform prescribing decisions, while solutions included constructing appropriate context-specific guidelines and improving evidence-based practices through input from local stakeholders, including surgeons, clinical pharmacists, and nurses.

**Conclusions:**

Study participants perceived that existing evidence and guidelines did not apply in their settings due to high rates of surgical site infections and gaps in perioperative IPC practices (e.g., availability of water for handwashing, sterility breaches). These gaps were a key contributor to prophylactic antibiotic overprescribing, reinforcing the need to strengthen upstream and perioperative surgical antisepsis processes. The findings of this study underscore the importance of engaging multidisciplinary teams in strengthening antimicrobial stewardship efforts, aligning processes to achieve compliance with best practices, and the need for rigorous, contextually appropriate studies from these settings to inform policy.

**Supplementary Information:**

The online version contains supplementary material available at 10.1186/s12913-024-11650-4.

## Introduction

Antimicrobial resistance (AMR) was estimated to be associated with nearly 5 million deaths in 2019, with the highest burden in sub-Saharan Africa [1–4]. Antibiotic prophylaxis, including postoperative prophylaxis, are a significant source of antibiotics prescriptions, making up one in six inpatient antibiotic prescriptions worldwide [5]. Notably, the World Health Organization (WHO) recommends against prolonged courses of postoperative antibiotic prophylaxis for preventing postoperative surgical site infections (SSI) [5, 6]. However, data informing WHO recommendations are largely derived from high-income countries (HIC) where SSI rates are low; in settings where SSI rates are high, especially in low resource settings, postoperative antibiotic use is common [5, 7–10].

Ethiopian national guidelines state that a single dose of prophylactic antibiotics should be given within 60 min prior to skin incision, and that a repeated dose of prophylaxis may be required for prolonged procedure or when there is significant blood loss, but postoperative prophylaxis is recommended for < 24 h [11]. A large prospective cohort study based in Ethiopia, Liberia, Madagascar, India, and Bolivia found that even in settings where SSI rates are high, prolonged postoperative antibiotic prophylaxis did not reduce SSI but was associated with prolonged length of stay (LOS) [12]. With the growing emergence of AMR, surgical initiatives to implement antimicrobial stewardship (AMS) programs in low and middle income countries (LMICs) are critical. There is a need to identify drivers of antibiotic prophylaxis overprescribing to ensure AMS programs identify and address the root causes of these behaviors.

A prior mixed-methods study at a large academic referral hospital in Addis Ababa, Ethiopia identified that physicians and pharmacists felt there was a need for stewardship practices, but perceptions of factors contributing to AMR were varied [13]. For example, pharmacists were more likely to link broad-spectrum antibiotics with AMR, but physicians were more likely than pharmacists to attribute lack of diagnostic tests to antibiotic overuse [13]. However, this study was not specific to surgical patients [13]. Additionally, a cross-sectional qualitative survey study of Ethiopian pharmacists found that pharmacists have positive perceptions towards AMS [14]. However, despite positive perceptions of stewardship programs, overprescribing of antibiotics is common [11]. There is a need to better understand this in surgical patients, as SSIs are a common and feared complication of surgery and may affect perceptions of the value of AMS within the surgical community. In our prior work, among surgical patients we found that over 90% of patients received postoperative antibiotic prophylaxis against WHO recommendations, with 28% receiving courses longer than 24 h [12]. We hypothesized that prolonged courses of prophylaxis were a response to high SSI rates in Ethiopia, which are reported to range from 11–23% [15–18]. In this study, we aimed to identify drivers of overprescribing of postoperative antibiotic prophylaxis, perceptions and barriers to stewardship, and perceived solutions.

## Methods

### Ethics approval

This study was approved by the Stanford University Institutional Review Board (IRB #69741) and St. Peter’s Specialized Hospital, where it was included as part of a larger study on antibiotic resistance in surgical care in conjunction with Addis Ababa University.

### Setting and eligibility

Participants were selected from three large, academic referral hospitals in Addis Ababa, Ethiopia: Menelik II Referral Hospital, Yekatit 12 Hospital, and Tikur Anbessa Specialized Hospital (TASH). Participants included attending surgeons, surgical trainees, and nurses who worked in the surgical department, with 6.91 mean years of experience, 6.66 standard deviation (see Table 1 for additional participant characteristics). All hospitals provide surgical care, including both general surgical care and subspecialty care in a broad range of fields (e.g., vascular surgery, neurosurgery, and plastic surgery).

**Table 1:** Demographics of Participants by Hospital

Characteristics	Menelik II (*n*)	Yekatit 12 (*n*)	TASH (*n*)	Mean years of experience in role (*n*, std dev)	Operative volume (*n* = mean cases/wk, std dev)
Total Participants	10	4	11	6.91^a^, 6.66	6.78, 3.27^b^
Female (nurses only)	0	0	5		
Role					
Attending Surgeon	6	4	2	8.36^a^, 8.63	6.04, 2.82
Fellow	0	0	2	1.5, 0.5	6.75, 0.75
Resident	0	0	2	3^a^, 0	11.25, 3.75
Nurse	4	0	5	6.78, 3.39	

^a^years of experience not available for two participants: one attending surgeon and one resident

^b^only surgeons included

### Interviews

We conducted a qualitative study consisting of semi-structured interviews from July to August 2023 in Addis Ababa, Ethiopia. We included surgeons from a variety of specialties, including general surgery, cardiothoracic surgery, vascular surgery, plastic surgery, pediatric surgery, neurosurgery, and hepatobiliary surgery, surgical trainees in these specialties, and ward nurses. Participants were selected by purposive maximum variation sampling to determine common patterns across a range of roles. Potential participants were accessible due to their familiarity with the research team members working at the hospital sites. Each participant was contacted in person or by phone by one of the Ethiopian authors, often TNM, or other members of the study team to discuss their willingness to participate. All potential participants were verbally informed about the aims of the study, that their names and affiliated hospital would be de-identified, and that participation was voluntary. All recruited participants agreed to participate, although in some cases they were not able to due to time constraints (*n* = 2). After verbal consent was obtained, interviews were conducted in-person at the hospital sites during clinical hours in English by one of the authors (SS), who was trained in conducting interviews. However, one of the authors (HA) performed Amharic translation and co-led interviews with nurses who preferred to speak in Amharic. We aimed to include at least 15 participants based on literature stating that 12 interviewees are generally needed to reach thematic saturation; sampling continued until data saturation was reached and no new themes emerged [19]. In total, 25 participants were recruited. A semi-structured interview guide was agreed upon prior to beginning the study and was used for each interview. Together, SS, MRN, TNM, and TGW developed and approved the interview guides for use (see Additional file 1 and 2: Antibiotics Qualitative Interview Guides). The interview guides were developed with consideration of the literature, prior qualitative studies done by our team in Ethiopia, and the context-specific expertise of the authors, particularly TNM [20]. The interview guides were designed to capture the rationale behind prolonged postoperative antibiotic prophylaxis, understanding of and challenges with following existing recommendations and evidence, and perspectives on antibiotic resistance in Ethiopia. The semi-structured interview guide allowed for focused questions for surgeons and nurses to reflect their different scope of practice and perspective within the care team. For the most part, interviews were similar as the study went on, with one major modification: questions regarding operating room (OR) sterility were too lengthy and the majority of themes were reached quickly in the interview. Several follow-up questions on OR sterility were removed from future interviews to keep interviews at an appropriate length and because saturation was reached in these areas.

Each participant was interviewed once without any financial incentive included. After informed consent and verbal permission, audio recordings were obtained on an encrypted device. They were then de-identified, labeled in the format “Surgeon 1, Hospital 1,” etc., and transcribed. Each transcription was reviewed and edited by SS to address any transcription errors prior to uploading to the qualitative analysis software Dedoose (Version 9.0.107 Los Angeles, CA: SocioCultural Research Consultants, LLC www.dedoose.com*).*

### Recruitment and procedures

We conducted 25 semi-structured interviews about postoperative prophylactic antibiotic use with Ethiopian surgeons and nurses across all three tertiary care hospitals. Of these, 16 interviews were conducted with surgeons, 12 of whom were attending surgeons, two were fellows, and two were residents. In this cohort, six surgeons were from Menelik II, six were from Yekatit 12, and seven from TASH (Table 1). Nine interviews were conducted with surgical nurses along with an Amharic interpreter when preferred by the interviewee. Of these nine interviews, five were from TASH and four were from Menelik II. Interviews took around 30 min to complete (range for physician interviews: 17 to 48 min; range for nursing interviews, including translation time: 17 min to 49 min), consistent with the evidence-based recommendations for lengths of qualitative interviews and keeping in mind the time constraints of participants [21].

### Qualitative coding and analysis

A codebook was inductively and iteratively derived and applied to transcripts from all sites [22–25]. The transcripts were coded starting with one transcript from each hospital site with the goal of capturing a diversity of ideas (SS, MN, AZ). Co-authors (TW, TNM) further developed and approved the preliminary codebook [26]. The codebook was then reapplied to previously coded transcripts. After revisions of codes and definitions, the coding team (SS, MN) performed an IRR test to assess reliability of consistent code application of codes to the ideas presented in the text, with an IRR goal of a minimum kappa of 0.7 [27]. Once consistent code application was achieved, all transcripts were coded. The primary coding team met for a final agreement process and thematic analysis [28]; the team did not have any significant disagreements during this process.

## Results

A total of seven themes emerged from the data: (1) Perceived risk of SSI, (2) Poor stewardship among multidisciplinary teams, (3) Poor infection prevention and control (IPC) practices, (4) Availability and affordability of antibiotics, (5) Effects of patient preference for antibiotics, (6) Constructing appropriate guidelines and role of evidence-based practices, and (7) Concerns related to antibiotic resistance.

Findings showed a range of typically antibiotic prescribing practices across each patient’s hospital stay. Findings have shown that surgeons commonly prescribed between 24 and 48 h of postoperative prophylaxis, with some (particularly attending surgeons with more years of experience) who more often endorsed giving longer courses of prophylaxis, while two surgeons (one hepatobiliary surgeon and one general surgeon) felt postoperative prophylaxis was unnecessary. Findings from nurses showed that most patients receive a minimum of 24 h of antibiotics. It emerged from the findings that clinical decision-making around antibiotics prescribing was left to the surgeon with feedback given to the surgeons from other clinicians if they did not agree with the treatment course, including feedback from both nurses and general practitioners. In Ethiopia, surgical patients on the ward are often cared for by interns, general practitioners, and surgical residents of different levels, under the supervision of an attending surgeon.

Seven surgeons reported that in some settings, clinical pharmacists are available. They may make written recommendations on antibiotic prescribing, but direct, verbal communication between clinical pharmacists and surgeons is rare. Consulting with pharmacists was not reported to be a routine part of their workflow, and some surgeons noted that while sometimes clinical pharmacists join on ward rounds, this is not common and does not typically affect how they prescribe antibiotics. Similarly, microbiologists were not mentioned as being involved in antibiotic decision-making.

**Fig. 1:**
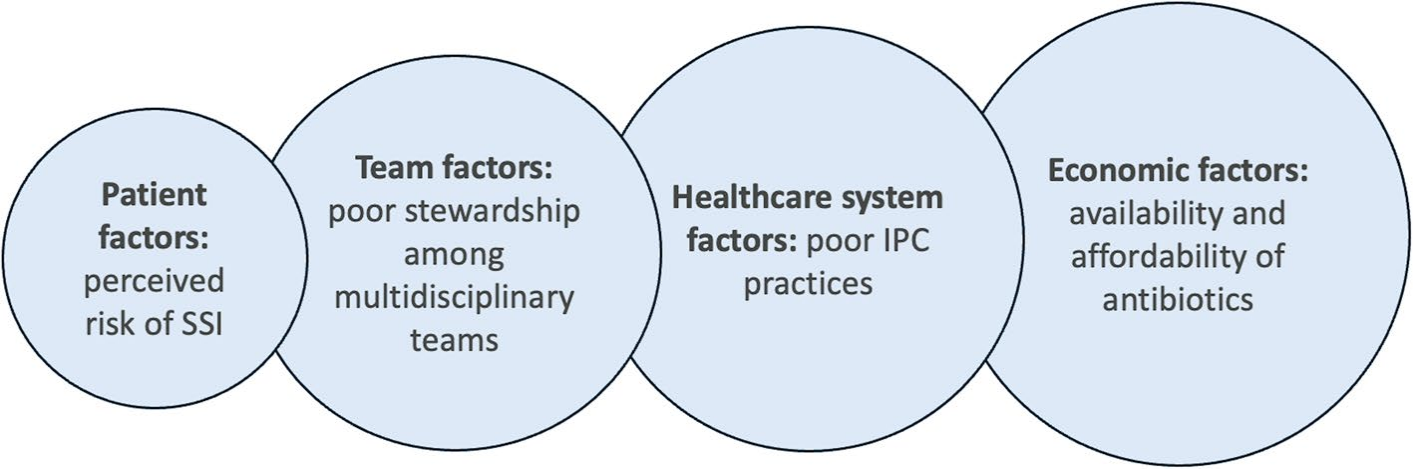
Schematic of Factors Contributing to Postoperative Prophylactic Antibiotics Overprescribing and Current Practices

When asked how the duration of postoperative antibiotic prophylaxis is determined, most surgeons stated it was multifactorial. Most surgeons stated they are aware of evidence-based recommendations to limit prophylaxis to a single dose of preoperative antibiotics but noted that actual prescribing behavior varied largely based on surgeon preference. Four levels of interconnected factors were identified as drivers of prescribing postoperative antibiotic prophylaxis: (1) Perceived risk of SSI as the individual/patient factor, (2) Poor stewardship among multidisciplinary teams as a team/community factor, (3) Poor infection prevention and control (IPC) practices within the hospital setting as a healthcare system factor, and (4) Availability and affordability of antibiotics as an overarching economic factor (Fig. 1; Table 2). A visual representation of these themes is presented in Fig. 1.

**Table 2:** Concerns Related to Antibiotic Resistance and Solutions

Concern	Solution
Antibiotics are overprescribed	• Controlled prescribing by provider status, with limited prescribing of some medications to senior physicians only
Poor infection prevention and control (IPC) practices and high surgical site infection (SSI) rates	• Minimize antibiotics resistance by strengthening IPC practices
Limited knowledge of resistance patterns	• Improved access to regional resistance patterns and development of hospital antibiograms
Evidence does not apply to local context	• Locally adapted guidelines • Studies based in the African context

### Theme 1: perceived risk of SSI

Surgeons expressed that they were more likely to prolong antibiotic prophylaxis when they perceived patients to be at high risk of having SSI. Nurses similarly reported a need for longer courses of postoperative prophylaxis due to concerns that short courses, such as those between 24 and 48 h, were associated with SSI.

*“The main problem here is*,* if the patient takes the prophylaxis for post-op*,* maximum of 24 or 48 hours and they are discharged with PO antibiotics*,* they will come back with infection after a week. So we come to the conclusion that it is due to the short-term of prophylaxis. I’m not agreeing with 24 or 48 hours of constant antibiotics*,* it’s not sufficient.” (Nurse 6)*.

Factors associated with higher risk of SSI included both patients’ clinical characteristics, such as immunocompromisation, as well as their ability to care for a surgical wound. For example, interviewees reported that poor patient hygiene and lack of access to reliable water supply upon discharge might motivate them to prescribe additional doses of postoperative prophylaxis. Additionally, proximity to follow-up care influenced decisions to prescribe postoperative prophylaxis. For patients with low socioeconomic status, particularly those coming to Addis Ababa from remote, rural areas, prescribing postoperative antibiotics was reported as a precaution to further reduce SSI risk, due to challenges accessing healthcare facilities or pharmacies and being able to afford medication in the event of an infection. Additionally, several surgeon interviewees expressed that more experienced surgeons are often inflexible in their practice patterns regarding antibiotics and are more likely to prescribe prolonged prophylaxis due to perceived risk of SSI.

*“If you don’t give prophylactic antibiotics*,* for ten patients*,* nine of them may be fine*,* but we are trying to avoid that one patient [having infection]…So another problem is in this government hospital*,* most of the patients who come here are from the rural region. So after you treat them*,* you will have to discharge them and they go to their hometown…In their hometown*,* the facilities are not that great. So you want to cover all bases and send them [with antibiotics]. So it may not be a rational use of antibiotics…almost all of us agree. But it’s just what we do.” (Surgeon 6)*.

A number of procedural characteristics were also perceived to be associated with increased risk of SSI, thus prompting prolonged courses of antibiotic prophylaxis. A long duration of surgical procedure, emergency operations, and use of a foreign body such as chest tubes, urinary catheters, or mesh were claimed as contributing factors leading to prolongation of postoperative antibiotic prophylaxis.

### Theme 2: poor stewardship among multidisciplinary teams

Clinicians from various backgrounds played different roles in contributing to antibiotic overprescribing. Although surgeons primarily reported making decisions on whether patients should receive postoperative prophylaxis, they endorsed getting feedback from general practitioners and nurses, who may question a decision to stop giving postoperative prophylactic antibiotics. Conversely, surgeons noted there was no one within the care team designated to champion stewardship efforts. Clinical pharmacists were identified as a potentially helpful resource in antibiotic decision making, but were not regularly available in most clinical settings. Neither surgeons nor nurses reported consulting with pharmacists to make decisions around antibiotic prescribing. However, surgeons and surgical trainees believed that having clinical pharmacists more readily available, with an increased role in the care team, would be helpful in improving antibiotic prescribing practices. In some cases the infectious disease teams are consulted for more complicated cases, but not for decision making surrounding postoperative prophylaxis.

*“So not only the patient*,* even the other health professionals…will question your judgment when you refuse to give antibiotics postoperatively.” (Surgeon 2)*.

Nurses identified their role as advocating for their patients, including regarding adequate postoperative antibiotics. They noted they might be the first to identify patients who received antibiotics beyond the expected duration and felt comfortable speaking up to clarify or correct errors.

*I have faced this kind of situation. When such a thing happens, immediately we communicate with the physician like, the patient was ordered antibiotics for 7 days, [but] he is taking for more than 9 or 10 days, so what was the reason and why not to stop or change some medication. (Nurse 6).*

### Theme 3: poor IPC practices

Both surgeons and nurses identified high rates of SSI due to poor IPC practices as a driver of antibiotic prescribing; both groups held beliefs that prolonging postoperative antibiotic prophylaxis would prevent SSI in their setting, especially in circumstances where OR sterility was in question. There was poor standardization in antibiotics prescribing courses because the standards are based on places with stronger IPC practices than their setting (Table 2). Although typically surgeons endorsed prescribing antibiotics for 24–48 h, if the surgeon identified concerns about the sterility of the operation, such as lack of clean equipment or running water, prophylactic antibiotics were often given for longer than 48 h to prevent infection. Surgeons identified additional concerns about poor IPC practices related to overcrowded ORs, a lack of appropriate sterile instrument reprocessing, and inconsistent access to running water which impairs ability to scrub properly. Whenever sterility was in question, surgeons reported compensating by prolonging postoperative antibiotic prophylaxis. Participants explained that antibiotics are often prescribed to “treat the mind” or cover the “not knowing” due to concerns about sterility in their OR environment. When asked about IPC practices, surgeons often stated unprompted that things were different in their setting than in HIC settings, and as a result they must give postoperative antibiotics even though it is not recommended. Surgeons felt that if IPC practices were strengthened, prolonged postoperative antibiotics may not be necessary; they noted they could abide by international guidelines if sterility was on par with that of HIC contexts.

*“We know in our setting it may not be appropriate. Because the primary thing you should do is infection prevention control (IPC)*,* which should be strong enough. In our setting*,* that’s not that much [valued]. To reduce infection you should use alcohol or in between patients*,* the water should be there. So you feel that you’re working in an unclean environment*,* and sometimes you need to use antibiotics to protect [against] it. So I don’t know if the setup in which WHO did the research actually represents ours. So if you feel you have strong infection prevention measures in your hospital*,* you feel that you’re safe not to use antibiotics.” (Surgeon 12)*.

*“Because*,* the [setting] here in our country… is not ideal. So even if you see the sterilization technique*,* the OR [environment]*,* even our nurse care and everything*,* it is usually substandard in our country. So to compensate for that*,* we just feel like the antibiotics will help us prevent possible complications*,* especially infections. So I do agree with it.” (Surgeon 6)*.

### Theme 4: availability and affordability of antibiotics

Access to a variety of antibiotics is limited in Ethiopia, with some antibiotics being either expensive, only available in privately owned pharmacies, or in some cases not available at all. Many clinicians expressed awareness that they are prescribing antibiotics that are different from what is recommended in the literature, but due to perception of limitations in access and cost, they have no alternative. Providers noted that ceftriaxone is often the option their patients can purchase, and in many cases is more available than narrow-spectrum antibiotics.

*“Sometimes we have to see what’s really affordable for that particular patient…Most of our patients mainly here in public hospitals…[can’t] afford expensive types of antibiotics… we don’t have any other choice than giving them for example ceftriaxone*,* that’s what we order… for all patients nowadays. Because previously*,* we were giving them ampicillin*,* we were giving crystalline penicillin*,* these medications are already out of the picture. What we have around is ceftriaxone. And that’s what most of our patients afford. Otherwise*,* there are others like ceftazidime*,* vancomycin*,* cefepime*,* meropenem… these are very expensive.” (Surgeon 3)*.

### Theme 5: effects of patient preference for antibiotics

Interviewees noted that patients are often worried if they are not getting antibiotics, or if antibiotics are stopped. According to surgeon interviewees, general practitioners and ward nurses were influenced by this communication from patients. However, surgeons stated that this patient preference does not prompt them to prescribe prolonged antibiotics. Surgeons try to explain why antibiotics are not necessary in certain cases, and this is usually met with understanding by patients.

*“Most patients… ask for [oral] antibiotics even after discharge. They think that a wound will heal only if you give them antibiotics. There is a strong request from the patient side for antibiotics. They think antibiotics are everything for wound healing. We try to convince them*,* but still the majority are not satisfied by the time they are discharged without antibiotics… they ask for [antibiotics] always… Why? Because most patients come from the rural setting*,* and low-level professionals in the rural setting*,* they treat everybody with everything*,* mild wounds even with antibiotics. So they have the wrong information that a wound without antibiotics will never heal.” (Surgeon 8)*.

### Theme 6: Constructing appropriate guidelines and role of evidence-based practices

While clinicians were often familiar with WHO guidelines and literature originating in HIC, most clinicians admitted that they were often not adherent to recommendations made based on data from those settings. Instead, they identified a preference to rely on studies done in the African context. Interviewees noted that SSI rates are higher in Ethiopia than in HIC, and WHO guidelines are not appropriate for the Ethiopian setting because OR sterility is challenging.

*“So when a study is conducted [on effectiveness of postoperative antibiotic prophylaxis on reducing SSI]*,* it should be in a setting as similar*,* at least as close to a setting as ours. So it should have an external validity*,* because if you see our surgical site infection rate in most of our settings*,* is close to 20 to 30%. So*,* irrespective of the type of surgery*,* you’re seeing*,* what most of these randomized trials are*,* their surgical site infection is around 2%. So it’s very difficult to take that evidence and use it in [our] setting. So we tend to use antibiotics for a little bit longer*,* you know*,* whether that is right or not*,* we have to understand it to refute it.” (Surgeon 2)*.

Although most surgeons stated there were no national guidelines, those familiar with the existing Ethiopian national guidelines similarly believed they are not applicable to the current setting. Several clinicians identified that mechanisms to enforce adhering with national guidelines are lacking.

Interviewees reflected that the guidelines that are taught in Ethiopia are mostly based on data from other countries that are typically higher-resourced. Most identified a need for local, regional, or improved national guidelines. Participants felt that guidelines need to take into consideration the availability of antibiotics and resistance profiles. They expressed the need for more studies in the Ethiopian or similarly resource-constrained contexts that experience high rates of SSI and face similar limitations in medication accessibility.

*“I would conduct a study in patients to continue with some kind of antibiotics for some time*,* or not by giving antibiotics postoperatively*,* as a prophylactic*,* and to do comparative study. My conclusion so far is not to give postoperative antibiotics when it’s a prophylactic [indication]. But that is not based on a study done in our [setting]…This is Africa*,* this is a different nature*,* a different psychological*,* a different cultural*,* different area*,* things*,* people*,* you know*,* everything is different… Or we would see you conduct a study with not giving antibiotics postoperatively and then see the outcome of the patient*,* see the complications*,* see the morbidity and mortality. This requires a prospective study with a good follow-up and one that takes a good number of patients so that we can come up with a better*,* tangible recommendation… Nationwide*,* we don’t have that guideline in the teaching hospitals. ” (Surgeon 3)*.

Nurses had differing views about practice adaptations that might address antibiotic resistance. Similarly to surgeons, they stated that clinical pharmacists would be valuable in the decision making process around antibiotic prophylaxis. Additionally, they advocated for surgical trained nurses to be staffing surgical units, as this is not always the case. However, unlike surgeons, nurses stated they do not see the need for major practice changes.

### Theme 7: concerns related to antibiotic resistance

The majority of surgeons and nurses identified antibiotic resistance as a significant public health problem. While two surgeons interviewed denied concerns about antibiotic resistance, most felt that it is a significant problem and were able to recall specific patients who grew cultures resistant to all available antibiotics. Many nurses had also seen patients resistant to all antibiotics and were concerned about this. Senior surgeons in particular noted a lack of control or regulation, stating that anyone can prescribe antibiotics, which contributes to overuse, while nurses specifically identified overprescribing of ceftriaxone, the most commonly prescribed antibiotic in surgical care, contributes to resistance. Clinicians also identified the lack of microbiology facilities for culture and sensitivity as a major contributor to worsening resistance. Often, clinicians felt that overuse of antibiotics was specifically a problem in private hospitals and clinics, but were less likely to identify it as a problem at their own facilities, all of which are public hospitals.

*“We are using ceftriaxone and the cephalosporins. But there was one study which was done in…the main hospital [here]… the study is shocking and says even more than 50% resistance to ceftriaxone. But since as I said*,* it is [easily] available to use and so we are still using it.” (Surgeon 15)*.

*“The practice is to write these prophylactic antibiotics upon discharge*,* for almost every patient. I doubt if this is right*,* because antibiotic resistance is really becoming a concern. I don’t know the last time where I ordered urine analysis and saw that it is sensitive to ciprofloxacin or to cephalosporins. I always see resistance to everything*,* [except] amikacin*,* gentamicin*,* and meropenem*,* just these ones are sensitive. So changing this practice is going to help*,* at least to save meropenem.” (Surgeon 6)*.

Surgeons believed they needed further studies to understand levels of antibiotic resistance, and that the public health implications should be given more attention. Several surgeons suggested limiting antibiotic prescribing by provider expertise might help reduce overprescribing, for example limiting the ability to prescribe some classes of antibiotics to fully trained surgeons. Participants called for a stronger working relationship amongst infectious disease, internal medicine, pharmacy, and microbiology to improve and standardize decision making.

*“I’d recommend if there is an AMS…or a clear guideline with the infectious disease team*,* the internal medicine person*,* the microbiologist*,* so that depending on the resistance pattern of the hospital…every professional will stick to that and prescribe and use the antibiotics rationally; otherwise*,* it will be difficult with the increased incidence of this antibiotic resistance.” (Surgeon 16)*.

Another suggestion was that institutions need access to regularly updated antibiograms to better understand the extent of resistance within their hospital settings. Finally, surgeons identified improved IPC practices might also limit antibiotic overprescribing and subsequently resistance. However, participants expressed that it can be difficult to change the practice habits of experienced surgeons and physicians. In order to do so, these individuals must be engaged in conversations around guideline development and practice patterns.

*“So we are trying to make the surgery safer by really [emphasizing] preparation of the patient and trying to make as clean [of a] surgery as possible… to minimize resistance*,* because we cannot afford expensive and new drugs. So availability and affordability are really an issue here.” (Surgeon 7)*.

## Discussion

The highest burden of AMS worldwide is thought to be in Sub-Saharan Africa [1]. Although participants commonly acknowledged prolonging antibiotic prophylaxis postoperatively despite recommendations against this practice, they also recognized resistance as a major public health problem. Both surgeons and nurses in this study shared personal experiences caring for patients who had infections that were resistant to all available antibiotics. One surgeon in particular expressed surprise to find widespread resistance to ceftriaxone, the most commonly used medication. Others noted that with limited availability of medications, conserving antibiotics is even more crucial.

This study identified key drivers of postoperative antibiotic prophylaxis (> 24 h) from a diverse group of hospital staff working in academic hospitals in Addis Ababa, including a high risk of SSI among patients coming from rural areas with poor infrastructure, a lack of multidisciplinary stewardship efforts, poor IPC practices, and medication availability. Although most surgeons acknowledged commonly prescribing antibiotic prophylaxis outside current international recommendations, which they are familiar with, AMR was identified as a major concern. Solutions offered included the development of guidelines accounting for local availability of antibiotics, resistance profiles, and basing practice changes on studies conducted in Ethiopia or similar settings. Several interviewees expressed that more experienced surgeons are often inflexible in their practice patterns, and that this would serve as a barrier to changing practices. Therefore, engaging these individuals in the creation of guidelines may be critical to ensuring guideline adherence. Evidence shows that engaging surgeons in AMS programming is important as they have the power to influence behaviors of the entire team [29]. However, the language surrounding the guidelines must match the priorities of surgical teams (e.g., focused on length of stay and outcomes) and guidelines should be implemented at convenient times, such as the surgical handover, where pharmacists could also participate in discussions [29]. In addition to guidelines, efforts are also needed to deal with failures in communication, encouraging distribution of responsibility for antibiotic decisions, and reducing fear of consequences from not prescribing [30].

A major theme identified was the use of antibiotics as a compensatory mechanism for poor perioperative IPC. While the WHO recommends against the use of postoperative prophylactic antibiotics use due to strong evidence that it has no benefit in preventing SSI [5, 6], here we found many clinicians believed that these recommendations, which are largely derived from data in HIC, do not apply in LMIC settings due to variable sterility practices and high SSI rates. These findings are consistent with other literature from LMICs, where the threat of SSI influences prescribing decisions more than the broader societal implications of antibiotic resistance [31]. While this sentiment was strong, several participants pointed to cases where they felt postoperative prophylaxis was unnecessary and not evidence based. These findings reinforce the need for initiatives to strengthen IPC practices and SSI prevention systems, which may have additional benefits in reducing antibiotic overprescribing in surgical patients. Although infection prevention standards are often not met in these settings, several studies have identified improvements are possible; for example, our own team has demonstrated the value of quality improvement programs to strengthen infection prevention standards [17, 18], while other study groups have shown the implementation of clean closure trays and routine glove changing can reduce SSI rates in this setting [32]. Further investments in such programs paired with strengthening of AMS protocols may offer one solution to improving AMR in these settings. This may also point to a need for physicians to communicate their perspectives on unnecessary postoperative prophylaxis with other team members, as it could contribute to both informal and formal practice changes. Evidence shows that hospitals provide a setting in which healthcare professionals can relate to each other, which can impact micro-level dimensions that then influence behaviors and everyday practice patterns [30].

Several participants pointed towards a need for identifying resistance patterns. More than 40% of countries in Africa do not have available data on resistance patterns [2]. Improving access to resistance patterns may improve awareness of antibiotic resistance, as data in Ethiopia is limited to several tertiary care centers. Additionally, local antibiograms may help improve targeted treatment of patients who do develop infections and reduce overuse of broad spectrum antibiotics; however, antibiograms are resource intensive to develop and maintain and early cessation of postoperative antibiotic prophylaxis, which is common in this setting, can be done without antibiograms.

In a qualitative study done in Kenya with 10 interviews with hospital managers who had knowledge and experience on AMS [33], several similar themes were identified in Kenya as in this study: the importance of AMS and the role of medicines and therapeutics committees, who set hospital guidelines, and availability of an antimicrobial formulary and usage surveillance systems [33]. While these interviews focus on hospital managers and high-level planning efforts for AMS, and less on motivations/drivers for the current state of antibiotics use as in the present study, we also found calls for increased communication about prescribing decisions among team members, and calls for resources such as strong guidelines, and improved infection prevention, all consistent with ideas relayed by interviewees in the present study. Calls for institutional guidelines are in keeping with findings from a prior study on AMR in Addis Ababa, but clearly barriers to implementing them still exist [34]. These studies, along with the present study, reiterate the importance of multidisciplinary approaches and infection prevention as core components of AMS initiatives. Furthermore, while a number of interviewees called for further research to be done via randomized controlled trials to inform the use of postoperative antibiotic prophylaxis in this setting, observational data from LMICs does not support its use for the prevention of SSI [12]. Along with implementing guidelines, improving education on postoperative prophylaxis in LMICs will be an important step forward and may address misconceptions that prolonged prophylaxis can compensate where IPC practices are poor. Even where data on resistance patterns and antibiotic use are limited, developing local guidelines may offer an opportunity to engage stakeholders in discussion about growing concerns of AMR, provide the opportunity to strengthen AMS standards, and promote accountability. Our team recently successfully piloted a quality improvement intervention that used hospital-based guideline development as part of a bundled intervention to promote education on postoperative prophylaxis in LMICs and engage surgical teams in evidence-based antibiotic prescribing practices [35]; further implementation and evaluation is ongoing.

Engaging with multidisciplinary teams has been shown to promote ownership of practice changes and sustainability of behavior change [10]. In this study, nurses often identified their role in advocating for discontinuing postoperative prophylactic antibiotics. However, nurses had different perspectives on the use of prolonged antibiotic prophylaxis. Many favored extending antibiotics for longer courses, believing current courses were inadequate in preventing infection, which may point to a gap in AMS knowledge among this group. Nurses also identified a lack of surgery-specific training, so further education on surgical infection prevention and AMS best practices may allow nurses to better engage in stewardship initiatives and understand evidence-based recommendations. Clinical pharmacists were also identified as important to creating accountability to AMS processes; however, in many cases they were not available or not involved in this capacity. Finally, because poor IPC practices were repeatedly identified as a driver of prolonging courses of antibiotics, hospital administration and leadership should also be engaged in order to identify ways to strengthen IPC systems. This also offers an opportunity for improved education, where surgeons who are familiar with evidence-based infection prevention practices can dedicate efforts towards strengthening those as an alternative to giving prolonged antibiotic prophylaxis. Our findings also underscore the need for engagement in stewardship efforts among different levels of hospital staff. As stewardship efforts are devised and implemented in settings like Addis Ababa, a wide variety of stakeholders should be engaged [36].

Finally, while several interventions have demonstrated the ability to strengthen IPC practices and improve AMS [17, 18, 31, 34], investment in such programs is needed. Policy changes at both national and international levels may also be warranted, as AMR continues to be one of the world’s most important public health threats [37]. 

### Strengths and limitations

We did not have the capacity to test the interview guide with subjects before use, which could have resulted in a better structure or important feedback. Interview participants were limited to academic referral hospitals in Addis Ababa and could have created a sampling bias. These hospitals are relatively higher resourced than district hospitals or hospitals located in more rural areas, and surgeons and nurses training or working in these hospitals often have specialty training and may be more likely to have personal experience in conducting research. However, they may be lower resourced than private hospitals, and conclusions made here may not be representative of practices there. Additionally, we used purposive maximum variation sampling, with a number of individuals selected for interviews who were acquainted with several authors and may have been more likely to participate based on their personal connections. However, it is common that many surgeons in Addis Ababa work at several hospitals and know one another. Notably, there were no female surgeons included in this study, which may reflect a shortage of women in the field in Ethiopia. One female surgeon was contacted to participate, but was unavailable at the times requested. This could also be partly due to using a purposive maximum variation sampling technique and knowing of fewer female surgeons to contact. However, it is possible the lack of female surgeons may influence the diversity of responses. Additionally, our sample was confined to surgeons and nurses, and it is possible that different healthcare professionals, such as clinical pharmacists and general practitioners, could have offered a different perspective. We were able to recruit 25 surgeons and nurses, and qualitative research methodology suggests this was sufficient to reach thematic saturation [19].

Furthermore, participants may have been affected by response bias based on the interview structure, or an inclination to respond in a way the interviewers were expecting. As much as possible, this effect was mitigated by the use of neutral phrasing of interview questions. As many participants were connected in some way professionally to team members, they may have been hesitant to freely express their views or felt pressured to give socially acceptable answers. In addition, nurses were interviewed accompanied by a translator who was a physician, and this may have biased their responses to be more positive towards physician leadership and multidisciplinary team dynamics. Additionally, the translator was not sworn or trained specifically for work as an interpreter, which may have impacted the accuracy of translation. Future studies should include a broader range of participants.

Apart from noted limitations, a concerted effort was made by the research team to sample from a wide variety of types of surgeons and nurses, in order to provide the best possible proxy of these professional communities. Care was taken to bring up findings from the interviews to the team as they progressed to revise interviews as necessary and ensure cohesiveness of data collected. Additionally, when ambiguities arose in the interviews, attempts were made by the interviewer to clarify points through follow-up questions. Any remaining ambiguities in the data were discussed by the research team during the coding process and were excluded from the analysis where appropriate.

The areas that we have highlighted are not only relevant to sites in Addis Ababa, but point towards the impact of context specific factors relevant in LMICs where variation in resources and culture are important considerations in implementing AMS quality improvement programs. While we cannot immediately generalize these findings outside of Ethiopia or beyond urban settings, they are consistent with the experiences of our authors who live and work in Ethiopia. Lastly, although other LMIC hospitals may have commonalities, there is still wide variability even within LMIC hospitals, which points to the need for similar studies to be done elsewhere [38].

## Conclusions

The findings of this study highlight the importance of poor IPC practices as a key contributor to antibiotic overprescribing. While implementing IPC practices in LMICs can be challenging, improvements have been demonstrated by improving adherence with infection prevention standards [17, 18] and implementing low cost interventions such as clean closure trays and routine glove changing [6]. These initiatives aimed at strengthening surgical antisepsis should be integrated into AMS initiatives at the regional, national, and interventional level, as surgical prophylaxis is a significant proportion of antibiotics prescribed globally. Emphasizing IPC strengthening may address the underlying cause of antibiotic overprescribing. Study participants identified a lack of standardization in determining who may benefit from prolonged antibiotic prophylaxis. In many cases, participants suggested that developing guidelines might strengthen stewardship practice and curb antibiotic resistance; in several cases they made these suggestions unprompted, early in the interview, prior to being asked targeted questions on these topics. However, many interviewees felt international guidelines failed to meet the challenges of the local context, and there was a marked knowledge gap regarding the national guidelines. Even within LMIC settings, prolonged postoperative antibiotic prophylaxis has not been shown to reduce SSI [12]; engaging stakeholders in the development of local, context-appropriate guidelines based on both international and locally-generated evidence may present one strategy to improve surgical AMS. Misconceptions about the utility of postoperative prophylaxis in SSI prevention was stated by a number of interviewees - this emphasizes a need for further education in this area. There is a need for continuous training informed by behavior change principles, partly to address the noted inflexibility of some of the more senior surgeons, and also nurses, on appropriate IPC standards and antibiotics utilization. The findings of this study point to a need to engage multidisciplinary teams in strengthening AMS efforts and guidelines in their settings, and increased efforts to investigate this area further, so that AMR is prioritized by policymakers on a national level. Further research applying behavior change, educational initiatives on AMS, and IPC strengthening to these settings is needed to curb AMR in this region.

## Supplementary Information

Supplementary Material 1.

Supplementary Material 2.

## Data Availability

De-identified data can be made available upon reasonable request to the authors.

## Abbreviations

SSI: Surgical site infections
IPC: Infection prevention control
AMR: Antimicrobial resistance
WHO: World Health Organization
IRR: Inter-rater reliability
AMS: Antimicrobial stewardship
OR: Operating room
LOS: Length of stay
LMIC: Low-and-middle income countries
TASH: Tikur Anbessa Specialized Hospital
HIC: High-income countries
