# Supplementary material for: Qualitative drivers of postoperative prophylactic antibiotics use and resistance in Ethiopia
Source: BMC Health Serv Res. 2024 Oct 22;24:1267. doi: 10.1186/s12913-024-11650-4 (PMC11495102; doi:10.1186/s12913-024-11650-4)
Supplement: Supplementary file 1 — Supplementary Material 1. [file 12913_2024_11650_MOESM1_ESM.docx]

# ANTIBIOTICS Qualitative Interview Guide

| Participant ID: |  |
| --- | --- |
| Date: |  |
| Interviewer Name: |  |
| Project Site: |  |
| Participant Role  (in Hospital): |  |
| Others present: |  |

## **Before starting:**

Emphasize that information will be de-identified & your names / name of hospital will not be used (even in publication)

[Once recording is on, again note that participation is voluntary, and that the participant agrees to the interview – verbal consent.]

*[intro: just trying to understand perspectives and where the gaps in research are, because we hope they can be filled]*

## **Questions**

## Participant Information

1. What is your role in the hospital?
2. How long have you worked in this hospital?

## BACKGROUND PERSPECTIVE AND training

1. Could you talk a little bit about any recommendations given in your nursing training for antibiotics use, and specifically for postoperative prevention of infection?
   1. Do you agree with these recommendations?
      1. If not, what would you prefer to do?
2. How long after surgery do you your patients continue taking prophylactic antibiotics?
   1. Does this vary with context (type of operation, patients, etc.)?
3. In some settings, patients consider IV antibiotics with good medical care. In your setting, if you don’t give a patient antibiotics, do they have concerns with that?

## TEAM DYNAMICS

1. Who decides when to stop postoperative prophylactic antibiotics? [surgeons, GPs, nurses, etc.]
   1. How are care decisions communicated?
   2. Are you engaged in the decision making in terms of reminding them to stop or change the antibiotics?
2. With respect to care decisions about continuing prophylactic antibiotics postoperatively, and more generally,
   1. Do you feel comfortable speaking up and advocating for treatment change within a care team when you feel it is necessary?

## pOTENTIAL changes

1. What is your perspective on antibiotic resistance patterns in your country, [and in your hospital if not previously mentioned]?
2. What types of changes, if any, do you think need to be made to antibiotics practices in your hospital?

**Is there anything else that you’d like to tell me about postoperative prophylactic antibiotics use at your hospital? Is there anyone in particular you think I should talk to at your hospital, or elsewhere, about this topic?**
