# Supplementary material for: Qualitative drivers of postoperative prophylactic antibiotics use and resistance in Ethiopia
Source: BMC Health Serv Res. 2024 Oct 22;24:1267. doi: 10.1186/s12913-024-11650-4 (PMC11495102; doi:10.1186/s12913-024-11650-4)
Supplement: Supplementary file 2 — Supplementary Material 2. [file 12913_2024_11650_MOESM2_ESM.docx]

# ANTIBIOTICS Qualitative Interview Guide

| Participant ID: |  |
| --- | --- |
| Date: |  |
| Interviewer Name: |  |
| Project Site: |  |
| Participant Role  (in Hospital): |  |
| Others present: |  |

## **Before starting:**

Emphasize that information will be de-identified & your names / name of hospital will not be used (even in publication)

[Once recording is on, again note that participation is voluntary, and that the participant agrees to the interview – verbal consent.]

*[intro: just trying to understand perspectives and where the gaps in research are, because we hope they can be filled]*

## **Questions**

## Participant Information

1. What is your role in the hospital?
2. How long have you worked in this hospital?
3. **[if OR team member]** How many operations did you participate in last week?
   1. What is the average # of operations you do in a week?

## BACKGROUND PERSPECTIVE AND training

1. Could you talk a little bit about the recommendations given in your training for postoperative prophylactic antibiotics use?
   1. Did these recommendations vary with context (type of operation, patients, etc.)?
   2. Do you agree with these recommendations?
      1. If not, what would you prefer to do?
2. How long after surgery do you continue prophylactic antibiotics [in wound class I/II]?
   1. Are there differences in how you would like to treat a patient with antibiotics postoperatively, and what is possible at your hospital?
      1. Are any medications restricted or hard to get?
      2. How does financial status of patients influence this?
3. [It sounds like practice patterns in Ethiopia are sometimes different from WHO. I’m trying to understand what makes your setting different, as you’re an expert in your setting]. The WHO recommends against continuing antibiotics postoperatively for the prevention of infection. Evidence used by WHO to make these recommendations does not show benefit in preventing surgical site infections by giving antibiotics after surgery.
   1. Can you tell me why you feel like these recommendations are appropriate for your practice setting?
   2. Why or why not?
4. What do you think about the Ethiopian national guidelines on prophylactic antibiotics?
   1. Do you follow them? Why or why not [are they appropriate to your practice setting]?
      1. [if not], what evidence do you use instead to make decisions?
5. Do you experience any challenges at your hospital with OR sterility? [if not described, prompt with respect to the following areas]:
   1. handwashing/skin preparation?
   2. surgical gown/drape integrity (sterilized / no holes)?
   3. preoperative antibiotic administration?
   4. sterility of instruments? (having indicators, verifying that autoclave is appropriately sterilizing, etc.)
   5. gauze counting?
   6. WHO Surgical Safety Checklist use?
6. **[if surgeon]** What clinical criteria do you use to decide when a patient should stop receiving antibiotics postoperatively for prevention of infection?
   1. [does this have to do with if patient is getting better, what you see in the OR, and/or data?]
   2. Are you happy/satisfied with the way that cultures are processed in your facility?
      1. If you take cultures, in which patients do you take them?
      2. Where are they processed?
      3. How often do you hear the results of these cultures? How long does it take to hear results?
   3. In some settings, patients consider IV antibiotics with good medical care. In your setting, if you don’t give a patient antibiotics, do they have concerns with that?

## TEAM DYNAMICS

1. **[for physicians]** With respect to care decisions about continuing prophylactic antibiotics postoperatively,
   1. Are there clinical pharmacists on you work with on your ward?
   2. Do you work with clinical pharmacists in making decisions about medication prescribing?
      1. If the pharmacist has an opinion on care, how do you get that information from them?
2. **[for non-physicians]** With respect to care decisions about continuing prophylactic antibiotics postoperatively, and more generally,
   1. Do you feel comfortable speaking up and advocating for treatment change within a care team when you feel it is necessary?

## pOTENTIAL changes

1. What is your perspective on antibiotic resistance patterns in your country, [and in your hospital if not previously mentioned]?
   1. What types of changes, if any, do you think need to be made to antibiotics practices in your hospital?
   2. What kind of evidence would you need to see to know whether it is safe to shorten the length of postoperative prophylactic antibiotics course?
   3. Is antibiotic resistance a topic of discussion among you and your peers?
      1. if so, what concerns have your peers mentioned, if any?

**Is there anything else that you’d like to tell me about postoperative prophylactic antibiotics use at your hospital? Is there anyone in particular you think I should talk to at your hospital, or elsewhere, about this topic?**
